# Structural and biochemical studies of mobile retrotransposon proteins in action

**DOI:** 10.1016/j.sbi.2025.103053

**Published:** 2025-05-06

**Authors:** Akanksha Thawani, Kathleen Collins, Eva Nogales

**Affiliations:** 1 California Institute for Quantitative Biosciences (QB3), Berkeley, CA, USA; 2 Department of Molecular and Cell Biology, University of California Berkeley, Berkeley, CA, USA; 3 Howard Hughes Medical Institute, Chevy Chase, MD 20815, USA; 4 Molecular Biophysics and Integrated Bioimaging Division, Lawrence Berkeley National Laboratory, Berkeley, CA, USA

## Abstract

Autonomous non-long-terminal repeat (non-LTR) retrotransposons, including long interspersed elements (LINEs), are mobile genetic elements abundant in eukaryotic species that shape the genomic landscape and host physiology in both health and disease. Non-LTR retrotransposons create new genomic copies through a mechanism termed target-primed reverse transcription, where the retrotransposon-encoded protein nicks target DNA to prime reverse transcription templated by bound RNA, typically its own encoding mRNA. Until recently, structural information on non-LTR retrotransposons was lacking due to challenges in purification and reconstitution of active complexes. Recent biochemical studies and cryo-electron microscopy structures of complexes from insect, bird, and turtle site-specific R2 retrotransposons and the human LINE-1 retrotransposon have provided important insights. Here we discuss these studies and their implications for retrotransposon evolution and eukaryotic genome biology.

Retroelements are mobile genetic elements that are widespread in all three domains of life [1,2]. In multicellular eukaryotes, the copy-and-paste mechanism of retrotransposons has generated a large fraction of genome content, shaping both the genetic landscape and host physiology [3–7]. Despite the inherent potential for conflict between retrotransposon mobilization and stability of the host genome, significant accumulation of retrotransposons has occurred during evolution [2,8]. The long interspersed element (LINE) retrotransposons that are common in mammals are a branch of the larger family of non-long terminal repeat (non-LTR) retrotransposons, which, combining autonomously mobile retrotransposons (the focus of this article) and their passenger, nonautonomous genome insertions (see Ref. [9] for review), compose a shockingly large fraction of many mammalian genomes (far above 20%) [3,8,10]. The LTR-retrotransposons and endogenous retroviruses that evolved from ancestral non-LTR retrotransposons are described in other reviews (see Refs. [11,12]).

Eukaryotic retrotransposons are ancestrally related to mobile group II introns (Figure 1a) found in bacteria, archaea, and in the mitochondrial and chloroplast genomes of some eukaryotes [13,14]. Group II introns are highly structured catalytic RNAs that can self-splice and reverse-splice into genomic loci [13,14]. Derived from this ancestry, eukaryotic retrotransposons are more divergent in their target sites in a genome, with some being highly selective for a target DNA, while others insert into loci that share only a short consensus sequence [15]. The relatively well-studied site-specific retrotransposon R2 inserts into the tandemly repeated ribosomal RNA gene loci transcribed by RNA polymerase I (the rDNA) within the genomes of nonmammalian multicellular animals [16]. Decades of biochemical and biological study established the R2 retrotransposon from the domestic silk moth, *Bombyx mori*, as the first reconstituted model system for investigation of non-LTR retrotransposon protein activities [16]. Recent work has expanded studies of R2 to other species (Figure 1b). In contrast to the conserved targeting specificity of R2, the human genome has one autonomously active retrotransposon, LINE-1 (Figure 1c) that inserts widely throughout our and other mammalian genomes [1,3]. By mobilizing LINE-1 mRNAs and also nonautonomously mobile short interspersed nuclear element (SINE) RNAs, the LINE-1 encoded enzyme (ORF2 protein, ORF2p) has generated a third of the human genome [3]. Ongoing LINE-1 retrotransposition is a source of genome evolution and genetic diversity, and it has an impact on aging, neurodegeneration, and human disease [1,3,6].

Eukaryotic non-LTR retrotransposons propagate via a shared mechanism termed target-primed reverse transcription (TPRT), in which nicking of one target DNA strand by the retrotransposon-encoded enzyme creates a primer for reverse transcription of the retrotransposon mRNA or other bound template RNA [10]. TPRT activity of the R2 retrotransposon enzyme was robustly reconstituted *in vitro* [17], but TPRT activity by that of the human LINE-1 ORF2p remained very low [18] until recent work [19]. Structural characterization of retrotransposon proteins and ribonucleoproteins had remained elusive, limited by biochemical challenges, particularly for the human LINE-1 system. In the last two years, however, cryo-electron microscopy (cryo-EM) has provided atomic-level insight into the TPRT mechanism of both eukaryotic R2 and human LINE-1 retrotransposons [19–24]. In this review, we first discuss the recent cryo-EM structures of insect and vertebrate R2 proteins and human LINE-1 ORF2p during the process of new DNA insertion to target DNA. We then highlight progress in our understanding of the biochemical steps in the mechanisms of retrotransposition and discuss their implications for genome biology and evolution. We finish with what we believe are outstanding questions about mechanism that remain to be resolved.

## Cryo-electron microscopy structures of R2 retrotransposon assemblies from insect and vertebrate species

R2 retrotransposons are site-specific elements that copy-and-paste into the tandemly repeated rDNA gene loci and exist within the genomes of multicellular animals, including insects, crustaceans, and nonmammalian vertebrates. The *B. mori* R2 protein, hereafter BoMo, has served as a model eukaryotic retrotransposon protein for TPRT. While prior biochemical work established that the R2 protein recognizes the 3′ untranslated region (UTR) of the retrotransposon RNA and nicks the first target DNA strand of a 28 S rDNA locus to initiate TPRT, the atomic-level underpinnings of TPRT initiation had remained elusive. Two recent cryo-EM studies [20,21] have now provided insights into retrotransposition by describing the structures of BoMo initiating TPRT bound to 3′ UTR RNA and target DNA (Figure 2a–d). Simultaneously, in efforts to engineer the R2-encoded proteins for transgene insertion into rDNA loci in human cells, we sampled the diversity of eukaryotic R2s and identified the A-clade R2 proteins from avian species, including *Taeniopygia guttata* (TaGu), to be highly efficient for precise transgene delivery [26,27]. In contrast, the D-clade BoMo was inefficient at transgene insertion in human cells [27]. More recently, we determined the cryo-EM structures of TaGu and an A-clade R2 protein from a turtle (*Platysternon megacephalum*, PlaMe) in distinct biochemical steps to provide further insights into TPRT [22] (Figure 2e–f).

Collectively, the different R2 structures inform us about mechanisms fundamental to TPRT, illustrating the nicking of a target DNA strand by the restriction-like endonuclease (RLE) and the transfer of the nicked 3′ end to the reverse transcriptase (RT) core, where it base-pairs with the 3′ UTR RNA to initiate first-strand synthesis (Figure 2c). Overall, all R2 proteins recognize the rDNA target through an extensive surface composed of the N-terminal DNA-binding zinc-finger and Myb domains and the C-terminal RLE domain (Figure 2c–d). All R2 proteins similarly engage with their cognate 3′ UTR RNA using an expansive surface consisting of the N-terminal extension (NTE) and RT domains (Figure 2a–d). A major difference between D-clade and A-clade R2 proteins lies in the number of N-terminal zinc fingers. Whereas the D-clade BoMo has one zinc finger and a Myb domain that interacts with a small surface area on rDNA upstream of the nick site, the A-clade TaGu and PlaMe have a longer upstream rDNA recognition surface consisting of three zinc fingers and the Myb domain (Figure 2d–e) [20,22]. The most N-terminal zinc finger (ZnF3) in A-clade TaGu and PlaMe also engages the 3′ UTR RNA (Figure 2c–d) [20,22].

**Figure 1:**
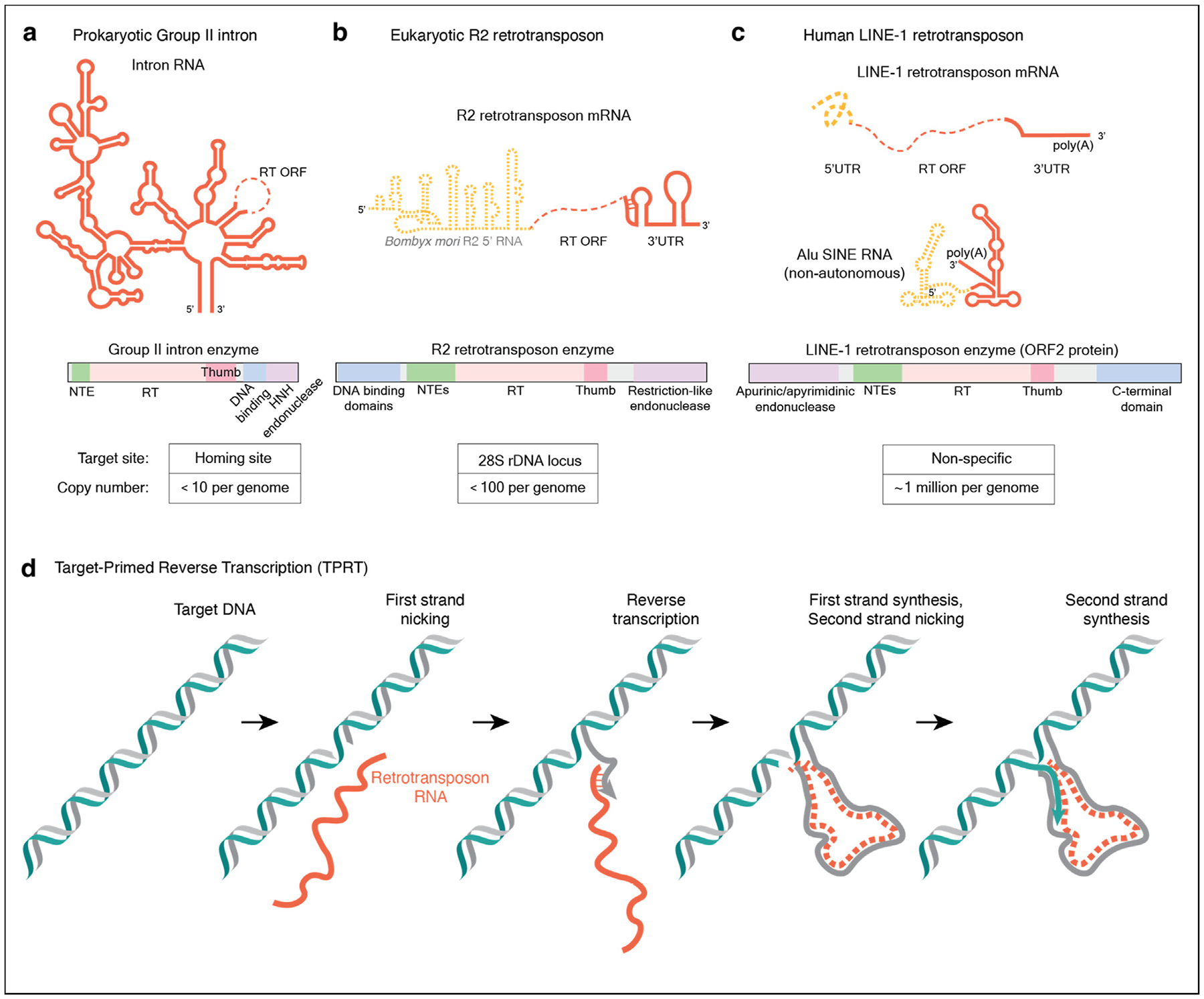
Autonomously mobile non-LTR retroelements in prokaryotes and eukaryotes. (**a**) Bacterial group II intron encodes a long, structured intron RNA (top) and an enzyme with reverse transcriptase and often endonuclease activities (middle). (**b**) Eukaryotic R2 retrotransposon inserts into rDNA loci and encodes an RNA with structured 5′ and 3′ regions (top) and an enzyme with endonuclease and reverse transcriptase activities (middle). The 5′ RNA illustrated is based on *B. mori* R2 RNA (ref: [21,25]) and may be unique to silk moth R2; the 3′UTR illustration is based on vertebrate A-clade R2 RNAs (ref: [22]), with many other R2s having a stem-loop in place of the pseudoknot. (**c**) Human LINE-1 retrotransposon inserts throughout the genome, copying LINE-1 and also nonautonomous SINE RNAs (Alu RNA is illustrated as one type of SINE). It encodes for a genomic RNA that gains a long poly(A) tail and translates ORF1p (not shown) and ORF2p (middle). The LINE-1-encoded enzyme ORF2p has endonuclease and reverse transcriptase activities. RNA features shown in solid red color in (**a**–**c**) are sufficient for new gene insertion. Below the schematics in (**a**–**c**), the tables illustrate the expansion of typical retrotransposon copy number per genome. (**d**) Biochemical stages of TPRT are illustrated with nucleic acids only. The removal of RNA from cDNA:RNA duplex is not well characterized, indicated by RNA conversion to a dashed line in the illustration. LTR, long-terminal repeat; UTR, untranslated region; LINE, long interspersed element; SINE, short interspersed element; ORF, open reading frame; TPRT, target-primed reverse transcription.

**Figure 2:**
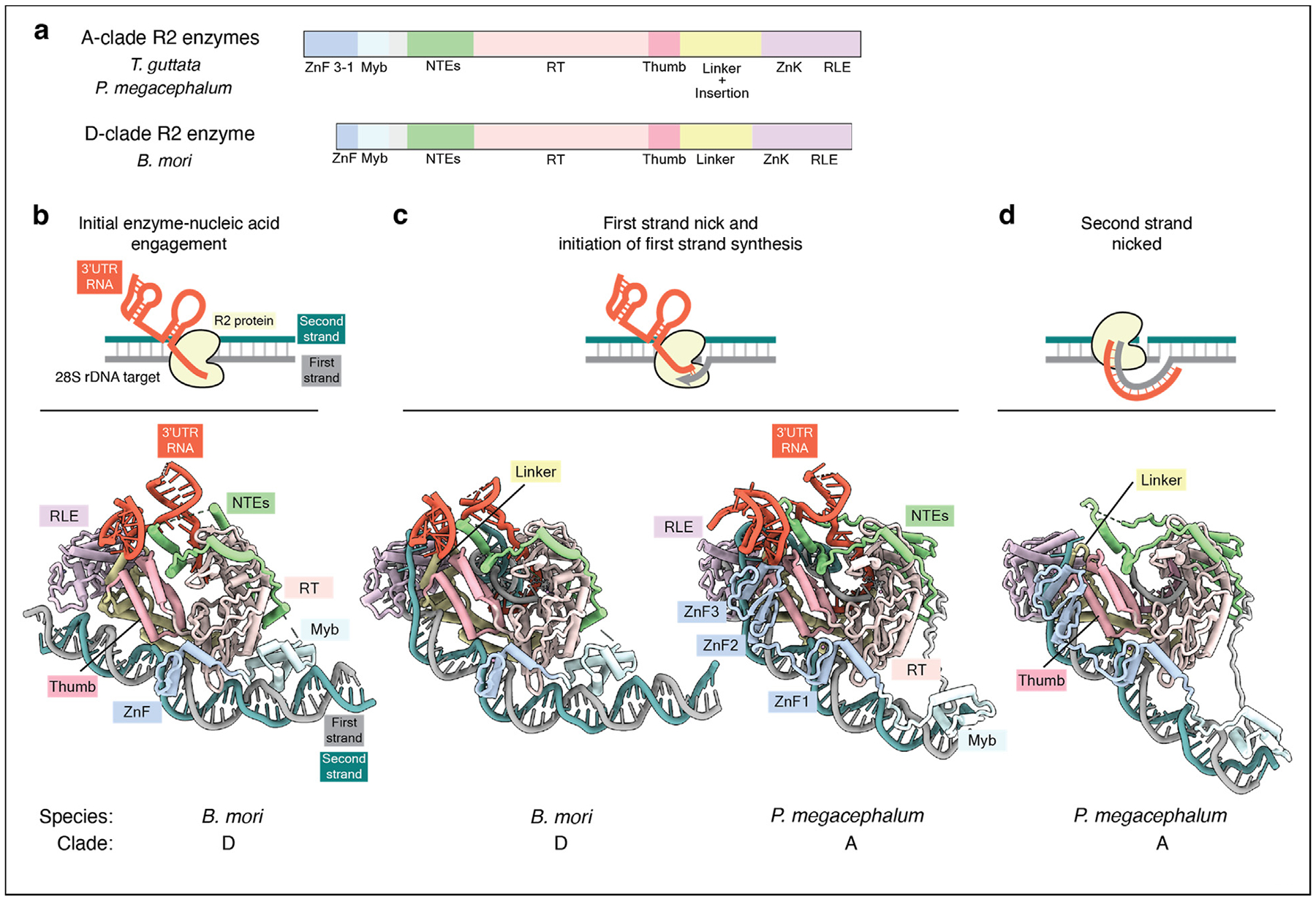
Cryo-EM structures of R2 retrotransposon assemblies during TPRT. (**a**) Domain architectures of A-clade and D-clade R2 proteins. ZnF, zinc-fingers; Myb, DNA-binding Myb domain; NTEs, N-terminal extensions; RT, reverse transcriptase; thumb, thumb domain; ZnK, zinc knuckle; RLE, restriction-like endonuclease. (**b**–**d**) Cryo-EM structures from three recent studies: initial enzyme-nucleic acid engagement for BoMo (PDB 8IBW) [21], first-strand nick and initiation of synthesis for BoMo (PDB 8GH6) and for PlaMe (PDB 9NL2) [20,22], and second-strand nicked state for PlaMe (PDB 9NL4) [22]. cryo-EM, cryo-electron microscopy; TPRT, target-primed reverse transcription; PlaMe, *Platysternon megacephalu*.

The R2 proteins are able to nick the second target DNA strand (Figure 1d). Second-strand nicking activity is suppressed in the presence of bound 3′ UTR RNA but becomes activated once the 3′ UTR RNA is removed from its initial binding site by first strand cDNA synthesis and production of the template RNA-cDNA duplex [21,22]. This finding suggests a sequential series of events, where second-strand cleavage occurs after first-strand nicking and cDNA synthesis (Fig. 1d, Figure 2b–d). *B. mori* R2 RNA contains a folded 5′ motif overlapping with the BoMo open reading frame (ORF) that may engage with BoMo to activate second strand cleavage [25]. Cryo-EM visualization of the protein-RNA interaction of BoMo and *B. mori* R2 5′ RNA shows that the 5′ RNA binds in a manner mutually exclusive with target DNA binding, including contacts with the BoMo surfaces that were bound to rDNA during TPRT initiation [21]. Notably, this 5′ RNA feature does not appear to be conserved in other species’ R2. Recently, we determined a structure of the A-clade PlaMe complex after second-strand nicking by mimicking the nucleic acid configuration when first-strand cDNA synthesis is complete (Figure 2d) [22]. Surprisingly, this structure revealed that the N-terminal zinc-fingers and Myb domain still bound to upstream target DNA in a similar configuration as the TPRT initiation complex (compare Figure 2d with the right side of Figure 2c) [22]. Upon second-strand nicking, the synthesis of a second cDNA strand has to occur to complete retrotransposition (Figure 1d), but the mechanistic details of this process are unclear.

## Biochemical and cryo-electron microscopy studies of human long interspersed elements-1 retrotransposition

**Figure 3:**
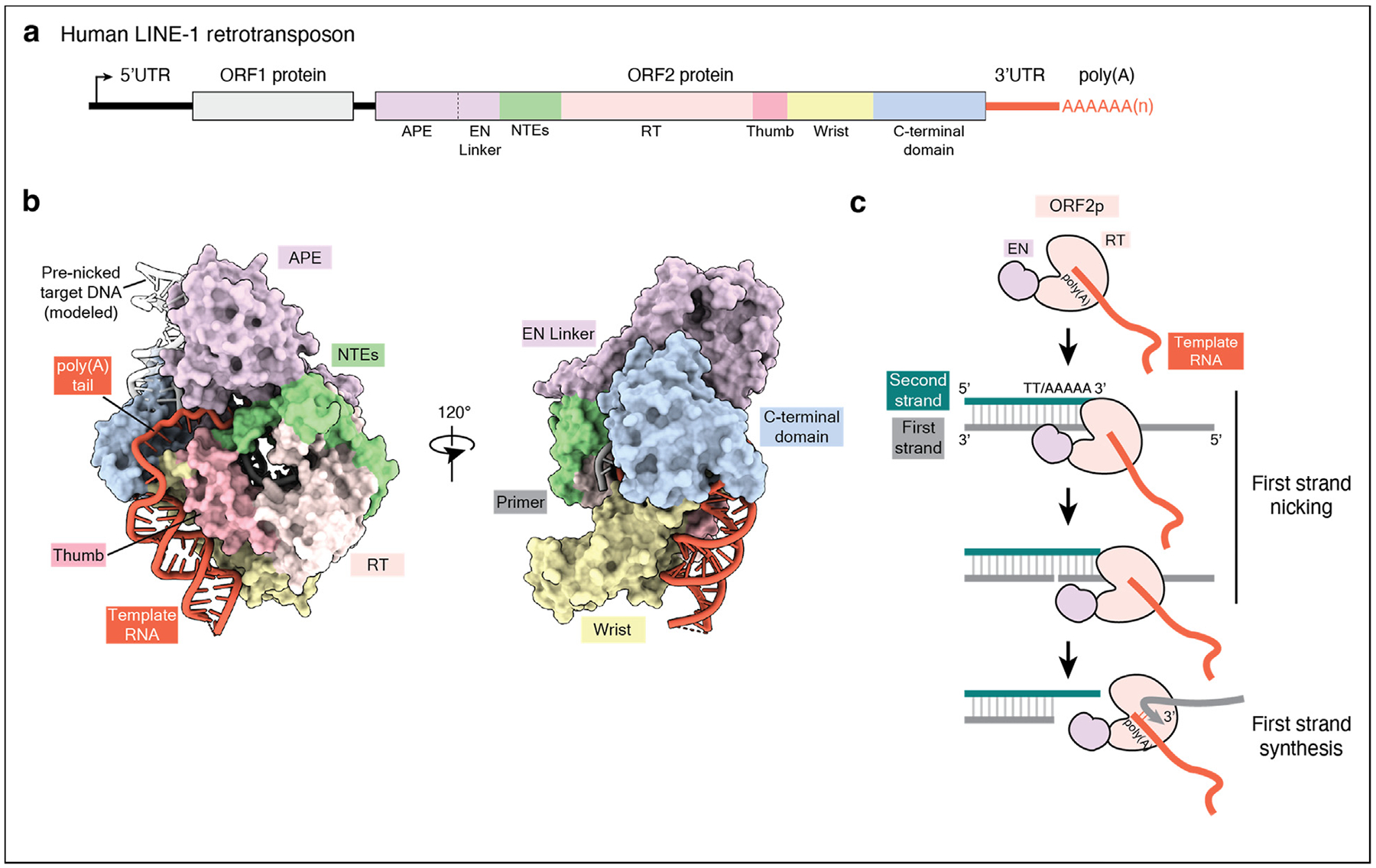
(**a**) Domain architecture of human LINE-1 retrotransposon, including a 5′ UTR region with RNA polymerase II promoter, ORF for RNA chaperone ORF1p, inter-ORF spacer, ORF for enzyme ORF2p, and 3′UTR terminating in a nongenomic poly(A) sequence. Domains of ORF2p (shown in different colors): APE, apurinic/apyrimidinic endonuclease; EN Linker, linker between APE EN domain and ORF2p core; NTEs, N-terminal extension motifs; RT, reverse transcriptase; thumb, thumb domain; wrist domain, which we previously referred to as RNA-binding domain [19]; and CTD, C-terminal domain. (**b**) Cryo-EM structure of ORF2p engaged with an Alu-like synthetic template RNA (PDB 8UW3) [19] shown in two orientations with protein in surface representation and nucleic acids in cartoon representation. Prenicked target DNA was modeled by superimposing the X-ray structure of prenicked DNA with APE domain (PDB 7N8S) onto the RNA-bound ORF2p structure [29]. (**c**) Cartoon illustrating that ORF2p carries out robust first strand nicking and TPRT only on DNA substrates with a 5′ single-stranded DNA overhang followed by double-stranded DNA containing the nick site [19]. UTR, untranslated region; ORF, open reading frame; EN, endonuclease; CTD, C-terminal domain; cryo-EM, cryo-electron microscopy; TPRT, target-primed reverse transcription.

The human LINE-1 retrotransposon encodes for two proteins: the RNA chaperone ORF1p and the retrotransposition enzyme ORF2p (Figure 3a–b) [3]. In contrast with the extreme target DNA selectivity of R2 proteins, ORF2p is relatively unspecific for target DNA due to the lack of sequence specificity of its DNA-binding domains. As such, LINE-1 insertions have degenerate target site specificity and insert widely throughout the human genome [3]. ORF2p has an N-terminal apurinic/apyrimidinic endonuclease domain (APE), homologous to enzymes involved in base-excision repair, that preferentially nicks a short consensus motif 5′ TTTTT/AA 3′ (Figure 3c) [28,29]. Understanding the biochemical and structural basis of LINE-1 retrotransposition has been limited by challenging purification and reconstitution of LINE-1 ribonucleoproteins, which in cells are scarce and have heterogeneously associated RNAs and other proteins [30–33]. A previous study aimed to reconstitute retrotransposition with overexpressed ORF2p, but the DNA nicking activity of this ORF2p was low, and robust TPRTwas not observed [18]. While X-ray structures of three specific domains of ORF1p and the APE domain of ORF2p had been previously reported [29,34–36], three recent cryo-EM studies have now provided structures of full-length ORF2p engaged with nucleic acids [19,23,24].

Using a bacterial expression system, Baldwin et al. purified an ORF2p protein core lacking the N-terminal APE domain and the C-terminal domain (CTD) and determined X-ray and cryo-EM structures of nucleic acid substrates bound in the ORF2p RTactive site [23]. In our own recent work, we purified full-length ORF2p overexpressed in large-scale insect cell cultures and used biochemistry and cryo-EM to provide new insights into LINE-1 retrotransposition [19]. We determined cryo-EM structures of ORF2p engaged with a structured native Alu SINE RNA 3′ end (Figure 1c) in early TPRT, with an RNA-DNA duplex enclosed within the ORF2p′s RT core (Figure 3b) [19]. The poly(A) tract of the template RNA engages with several domains, including the N-terminal extension motifs (NTEs) and preceding APE endonuclease domain (EN) linker, as well as the CTD, creating sequence-specific contacts [19,37]. We also showed that a stem-loop within an Alu or synthetic RNA is positioned by electrostatic interactions with ORF2p (Figure 3b) that would ideally locate an Alu RNA poly(A) tail for TPRT initiation [19].

Using biochemical analyses of TPRT, we observed that the DNA nicking and TPRT activities of ORF2p were greatly stimulated only when the TTTTT/AA nick site was positioned near the 5′ end of the target double-stranded DNA (dsDNA), but not when longer dsDNA was added upstream of the nick site [19]. A single-stranded DNA (ssDNA) region of 27 nucleotides (nt) placed 5′ of the target dsDNA nick positions was preferred, with much less stimulation by shorter ssDNA overhangs [19]. By superimposing the structure of APE domain co-crystallized with dsDNA [29] onto our full-length ORF2p structure, we deduced that the source of this preference could be a steric clash between the ORF2p CTD and dsDNA upstream of the nick site [19]. Confirming our findings, a subsequent study could detect no TPRT activity on fully duplex target DNA and detected TPRT using target DNA with a minimal 7 nt ssDNA 5′ overhang [24]. Thus, our findings show that ORF2p performs TPRT on DNA substrates that are structure-specific for a 5′ ssDNA overhang, an architecture that would be abundant on the primed lagging-strand template behind DNA replication forks. This result, along with the reported interaction of ORF2p with proliferating cell nuclear antigen (PCNA) [30], provides a molecular explanation for previous work demonstrating that LINE-1 retrotransposon insertion occurs preferentially during the S-phase of the cell cycle [38] and shows strong insertion preference for the lagging strand template [39,40].

Recently Ghanim et al. determined cryo-EM structures of ORF2p engaged with target DNA nicked on both strands [24]. Input DNA was prenicked on the first strand, but only a trace amount of upstream duplex DNA nicked also on the second strand was bound by ORF2p [24]. The nicked second strand density appears connected to an unassigned single-stranded RNA density that further connects to the template RNA in ORF2p′s RT active site [24], altogether forming the path of the poly(A) RNA tail in our cryo-EM structure [19]. Since ORF2p cannot make the first nick on a double-stranded target DNA [19,24], it will be important to understand the physiological relevance in duplex DNA binding in this configuration [24]. It will also be important to distinguish between the different modes of ORF2p binding to PCNA, which could occur via the originally defined motif on ORF2p [30] or a newly identified PCNA binding site with AlphaFold3 predictions [24]. There are many states of ORF2p nucleic acid interaction required in RNA selection, target site selection, nicking, and cDNA synthesis; understanding the transitions between these states will be highly informative for placing structural snapshots in context.

## Evolutionary aspects of retrotransposition mechanisms

When comparing the retrotransposition mechanisms of prokaryotic and eukaryotic non-LTR retroelements, a few key similarities and differences become apparent. While proteins from both systems use a DNA nicking event to prime reverse transcription of the retroelement RNA, the substrates they use are different. Eukaryotic retrotransposon RNA structures have become less complex (Figure 1a–c), concurrent with the change to a noncatalytic role. While a group II intron RNA adopts a complex folded structure [13,41–44], RNA structural complexity is reduced for eukaryotic R2 transposons [20–22], and further still for human LINE-1 due to ORF2p primary recognition of poly(A) sequence [19,37]. Concurrently, retroelement-encoded proteins have increased in number, size, and domain complexity, as has the role that the encoded proteins take in defining target DNA specificity of TPRT [19–21,45,46]. Notably, specific group II introns utilize the lagging strand DNA template and Okazaki fragments that are generated by DNA replication forks for insertion [14,47], paralleling human LINE-1 insertions [19,39,40]. In fact, many prokaryotic mobile elements harness vulnerabilities on the lagging-strand, including the exposed 3′ OH on the Okazaki fragment primer, exposed ssDNA, or the presence of replication-associated proteins as the key features for their targeting [48].

## Conclusions and outstanding questions

The recent cryo-EM and biochemical studies of insect and vertebrate R2 retrotransposon proteins and the human LINE-1 ORF2p have greatly advanced our structural and functional understanding of the insertion mechanisms of eukaryotic non-LTR retrotransposons. These studies have provided the necessary framework for further work aiming to fill the remaining gaps in our understanding of eukaryotic non-LTR retrotransposon mobility. Particularly, how an active retroelement RNA:protein complex is assembled for competent retrotransposition remains unknown. This process is presumed to involve cotranslational assembly of retroelement RNA:protein complexes in a mechanism termed *cis*-preference [49,50], but the generality of this human LINE-1 ribonucleoprotein assembly specificity remains to be determined [14]. Further, the role(s) of ORF1p in the LINE-1 retrotransposon process remain uncertain; ORF1p is thought to function as an RNA chaperone for LINE-1 mRNA, yet it is not essential for Alu RNA retrotransposition [51,52]. Finally, how DNA lesions created during retrotransposition are repaired to produce a stably inserted new retrotransposon copy also remains elusive. Future biochemical, functional, and structural work will elucidate these key steps for eukaryotic retrotransposition mechanisms.

## Data Availability

Data used is available on Protein Data Bank (PDB)
